# Harm reduction and abstinence-based models for treatment of substance use disorders during the COVID-19 pandemic: a global perspective

**DOI:** 10.1192/bji.2022.1

**Published:** 2022-08

**Authors:** Venkata Lakshmi Narasimha, JL Butner, Enjeline Hanafi, Mehdi Farokhnia, Roshan Bhad, Fatemeh Chalabianloo, Christos Kouimtsidis, Alexander Baldacchino, Shalini Arunogiri

**Affiliations:** 1 Assistant Professor, Department of Psychiatry, All India Institute of Medical Sciences (AIIMS), Deoghar, Jharkhand, India; 2 Addiction Medicine Specialist, Yale University School of Medicine, New Haven, Connecticut, USA; 3 Department of Psychiatry, Addiction Psychiatry Division, Faculty of Medicine, Universitas Indonesia, Dr Cipto Mangunkusumo General Hospital, Jakarta, Indonesia; 4 Staff Scientist, Department of Mental Health, Johns Hopkins Bloomberg School of Public Health, Johns Hopkins University, Baltimore, Maryland, USA; 5 Associate Professor, National Drug Dependence Treatment Centre, Department of Psychiatry, All India Institute of Medical Sciences, New Delhi, India; 6 Senior Consultant in Addiction Medicine and PhD Fellow, Department of Addiction Medicine, Haukeland University Hospital, Bergen, Norway; 7 Honorary Senior Lecturer in Addictions, Department of Medicine, Division of Brain Sciences, Imperial College London, United Kingdom; 8 Medicine, Psychiatry and Addictions Professor, Division of Population and Behavior Sciences, Medical School, University of St Andrews, UK; 9 Monash Addiction Research Centre and Eastern Health Clinical School, Faculty of Medicine, Nursing and Health Sciences, Monash University, Melbourne, Victoria, Australia. Email: shalini.arunogiri@gmail.com

**Keywords:** Abstinence, addiction, COVID-19, harm reduction, substance use, treatment

## Abstract

The COVID-19 pandemic has significantly affected treatment services for people with substance use disorders (SUDs). Based on the perspectives of service providers from eight countries, we discuss the impact of the pandemic on SUD treatment services. Although many countries quickly adapted in provision of harm reduction services by changes in policy and service delivery, some went into a forced abstinence-based strategy. Similarly, disruption of abstinence-based approaches such as therapeutic communities has been reported. Global awareness is crucial for responsible management of SUDs during the pandemic, and the development of international health policy guidelines is an urgent need in this area.

Harm reduction is an umbrella term used for a set of ideas, interventions and practical strategies aimed at reducing negative consequences associated with substance use and other health behaviours.^1^ Although the concept of harm reduction has existed for a long time, its formalisation started during 1980s in the context of the HIV epidemic, propelled by opioid use among those with injecting drug use, and later expanded to many other substances. In the context of opioids, the evidence-based harm reduction strategies include opioid agonist treatment, needle and syringe programmes, safe injection rooms, overdose prevention programmes, fentanyl strips, identification and treatment of sexually transmitted diseases, outreach and education. Such interventions have been found to be highly effective and remained the mainstay of treatment for opioid use disorders. Abstinence refers to complete cessation of substance use. The DSM-5 criteria for substance use disorders (SUDs) uses cut-offs of 3 and 12 months for early and sustained remission respectively. In general, abstinence-based models have dominated treatment programmes globally and have been an inherent component of residential programmes (e.g. therapeutic communities) and twelve-step facilitation.

The COVID-19 pandemic brought unprecedented challenges for individuals with SUDs and health professionals involved in their care. There were changes related to policy, availability of substances, patterns of use, substance-related complications and the provision of treatment services. In this brief review we discuss changes in harm reduction and abstinence-based approaches during the COVID-19 pandemic, based on perspectives from health professionals in eight countries and supplemented by data from global surveys of professionals involved in substance use treatment services conducted during the pandemic.

## Impact of COVID-19 on treatment services

During the COVID-19 pandemic, people with SUDs and their access to services were significantly affected across the world (Table 1).^2^ Aglobal survey during the initial months of the pandemic by the International Society of Addiction Medicine Practice and Policy Interest Group (ISAM-PPIG) observed a worldwide impact on SUD treatment and harm reduction services. Importantly, professionals in one-third of countries reported a shortage of methadone and buprenorphine supplies, and around 40% of countries witnessed a rapid decrease in harm reduction services, especially needle and syringe programmes and condom distribution. Professionals in more than half of the countries reported an impact on overdose prevention services, and around 80% reported that outreach services were affected.^4^ Service providers in low-income countries described significant impacts due to sudden policy changes, lack of transportation, reduction of emergency room beds and disruption of out-patient services.^5^ A second global survey by ISAM-PPIG conducted later in the pandemic observed changes in drug availability, safety and consumption patterns in early phases of lockdown due to COVID-19. Many countries reported reductions in drug supply, with an increase in the illicit market price of drugs. During the pandemic, some countries experienced an increase in fentanyl use among those on opioid agonist treatment and reported an increase in fentanyl-related overdoses.^6,7^ Overall, while the consumption of alcohol, cannabis, prescription opioids, and sedatives increased, there was a reduction in use of amphetamine, cocaine and illicit opiates.^5^ The European Monitoring Centre for Drugs and Drug Addiction (EMCDDA) also reported reduced activity in treatment services the during first 2 months of the pandemic and normalisation after June 2020.^8^ Additionally, it noted an increase in treatment demand, posing challenges for healthcare services. Similarly, access to abstinence-based approaches was disrupted as there was a reduction in the number of service providers and longer waiting times to access services.

**Table 1:** Changes in harm reduction and abstinence-based services across eight countries

Country	New harm reduction services introduced	Changes to existing harm reduction services	Changes to abstinence-based services
Australia^3^	Tele-health services Home delivery of OAT Increased roll-out and uptake of long-acting injectable buprenorphine	Expansion of harm reduction services – education on risks of unintended withdrawal Funding and support for naloxone provision Reduction in access to residential treatment owing to capacity limits secondary to physical distancing, increased waiting lists for detoxification/withdrawal and rehabilitation beds	None
Greece	Existing street work services included basic needs such as water, food and COVID-19-related hygiene products First ever shelter for homeless people who use drugs in Athens and Thessaloniki	Changes in OAT, with easier access and extended period of take-home medication	Provision of COVID-19 self-isolation facilities within residential rehabilitation units
India	Tele-health services	Low threshold for OAT initiation Increased duration of take-home OAT Take-home methadone was given for first time during lockdown Special 70% tax on Alcohol (labelled as 'special corona fees') Disruption of SUD treatment services across the country	Forced abstinence due to ban on alcohol and disruption of services for take-home buprenorphine Videos on handling withdrawal due to unavailability of alcohol and tobacco
Indonesia	Tele-health services for existing patients but not new patients For people with substance use disorder, delivery of anti-retroviral therapy to the person's house	Reduced harm reduction services Closure of many harm reduction centres and other addiction service centres Several methadone clinics were moved outside primary healthcare Addiction healthcare providers were empowered in COVID-19 health services Take-home OAT was extended from daily to twice weekly	Limitation or elimination of visiting hours at in-patient services Mandatory COVID-19 testing for admission
Norway	Home delivery of OAT Establishment of in-bed services for admission of patients in quarantine or isolation to reduce withdrawal symptoms and complications Use of telephone and digital consultations in follow-up of the most vulnerable patients with psychiatric comorbidities	Most of the existing harm reduction services expanded, e.g. increasing supply of clean syringes and user equipment; however, many low-threshold social rehabilitation and municipality care services were closed National guidelines specific on OAT emphasised lowering the threshold for OAT initiation and outreach delivery of medications Guidelines on the care of people with SUDs were issued to ensure responsible management of affected patients by assessing the need for temporary substitution with opioids, benzodiazepines or central stimulant agents under quarantine and isolation	No new abstinence-based models were introduced; the supply of alcohol was relatively stable during the lockdown period, although some restrictions were applied Some components are added to harm reduction strategies, e.g. considering temporary substitution treatment with prescribed medications such as opioids and benzodiazepines to reduce the withdrawal complications and to avoid infection spread by supplying the drugs needed under quarantine and isolation
Scotland	Home provision of methadone and buprenorphine by third-sector staff to individuals who were shielding Introduction of injectable buprenorphine	Expanded rapid provision of take-home naloxone	None
UK	Legislation for provision of accommodation to homeless people Telephone and digital provision of services, including group interventions	New guidance on treatment of alcohol dependence Some local policies made provisions for alcohol supply to those unable to purchase alcohol Increased periods of take-home OAT Relaxed norms for supervised consumption of OAT	Access to in-patient detoxification and residential rehabilitation services have been reduced, with longer waiting times mostly due to capacity restrictions
USA	Tele-health mediated initiation of buprenorphine	Low threshold for OAT initiation and longer duration of take-home medications	None

OAT, opioid agonist treatment; SUD, substance use disorder.

### Harm reduction services

Most countries quickly adapted to the unprecedented conditions imposed by the COVID-19 pandemic by moving to telemedicine-based delivery of services. During the pandemic, most countries also lowered the threshold for opioid agonist treatment (OAT) initiation and increased the duration of OAT (such as methadone and buprenorphine) dispensed. Home-based delivery of OAT was initiated by countries such as Australia, Norway and Scotland (UK).

In the USA, a major change during the pandemic was amendment of regulations to exempt physicians from certain certification requirements (commonly known as X-waiver) needed to prescribe buprenorphine for the treatment of opioid use disorder. Further, buprenorphine can be initiated via telehealth, but methadone maintenance treatment still needs to be started in person. During the pandemic, the USA piloted managed alcohol programmes (MAPs) among homeless individuals with severe alcohol use disorder to aid adherence to COVID-19 protocols. This study provided a foundation for expansion of MAPs as a recognised public health intervention for those unable to stabilise within existing care systems.^9^

During the pandemic, Australia increased roll-out and uptake of long-term injectable buprenorphine as a means of increasing access and convenience and decreasing travel-related risks. In Norway, harm reduction measures were strengthened by increasing access to clean syringes and equipment and lowering the threshold for OAT. Some countries increased the availability of needles from vending machines, increased provision of take-home naloxone, and increased funding and support for naloxone provision. At a national level, many countries, such as India, the UK, Australia and Norway, formulated guidance documents that included management guidelines, information and education materials related to SUDs.^10^ In Indonesia, anti-retroviral drugs for people with SUDs were delivered to their residences. In Indonesia, addiction specialists were deployed for COVID-19 care, resulting in considerably fewer healthcare professionals available to service patients with SUDs. With the prolongation of the COVID-19 pandemic and advent of further waves, harm reduction services continued to be significantly affected.

### Abstinence-based services

In many countries SUD treatment centres, including rehabilitation centres and those linked to general hospitals, were closed as they were considered non-essential.^2^ In Australia, because of social distancing regulations and requirements, there was reduction in overall bed numbers and an increased waiting list to get into treatment. In Indonesia, financial problems during the COVID-19 pandemic also led to lack of medication adherence because it was difficult for some patients to reach the new treatment centres. Integration of patients’ data as a unified system is still limited in Indonesia, so the new treatment centres had to reassess the patient, disrupting continuity of care. Reintegration of patients from rehabilitation services into the community also became problematic because the involvement of family and the community during the in-patient treatment or rehabilitation was limited.

The EMCDDA reported a disruption to therapeutic communities and to those in aftercare settings as unemployment increased during the lockdown.^8^ Scotland (UK) and Greece made legislative changes to provide shelter for homeless people, one of the most vulnerable populations with SUDs.

Although in many countries, alcohol was included as part of ‘essential’ goods and supply was maintained throughout the pandemic, in other countries, such as India and South Africa, the sale of alcohol was banned during the COVID-19 lockdown.^11^ In India, such a forced abstinence resulted in complicated withdrawals among people with alcohol use disorder (the forced abstinence model) but access to treatment for alcohol-related emergencies was not possible because of the lockdown.^12^ Vulnerable populations, especially those at risk of homelessness and those of lower socioeconomic status, went into unwanted abstinence due to disruption of services. In some countries, patients on OAT and benzodiazepine prescriptions who needed to be admitted to COVID-19 isolation facilities but were unable to access SUD services owing to the risk of contagion were advised on self-tapering regimens to avoid severe withdrawals. Owing to lack of availability of harm reduction services in isolation facilities and hospitals, many feared forced abstinence if they tested positive for COVID-19 and therefore some avoided testing. These unprecedented public health measures had an impact on social care systems, limiting the essential services to individuals with severe SUDs and, in some cases, widening extreme social inequities such as poverty and homelessness.^2^

## Summary

The COVID-19 pandemic has significantly affected people with SUDs because of lockdowns, sudden policy changes and disruption of treatment services. However, countries adapted quickly in provision of harm reduction services by initiating tele-health services, home-based delivery of medication, lowering the threshold for OAT initiation and relaxing the prescribing norms for opioid substitution medication, increased roll-out of long-term injectable buprenorphine and increased access to clean syringes. In several countries, guidance documents for healthcare professionals on the management of SUDs during the pandemic and adequate information for the general population have been made available in the public domain. Some people with SUDs went into unwanted abstinence due to policy changes and disruption of services. With the prolongation of the pandemic and subsequent waves, substance use treatment services continue to be affected drastically. Finally, the effects of the COVID-19 pandemic along with ongoing SUD epidemic synergistically resulted in a syndemic across the world. Therefore, it is important to identify and develop country- or region-specific strategies to mitigate the effect of the ongoing pandemic on people with SUDs. Global awareness is crucial for responsible managing of SUDs during the pandemic, and the development of international health policy guidelines is an urgent need in this area.

## Data Availability

Data availability is not applicable to this article as no new data were created or analysed in this study.
